# In Memoriam

**Published:** 1889-03

**Authors:** 


### IN MEMORIAM.

Why do you weep? Why rend the air with cries?
All men must die; and whether now or then,
Or when they may, what matter? Man but dies,
And dying, lives, and knows not death again.

If death is life eternal, purified
And purged from pain, cease then your causeless plaint;
He found life's greatest glory when he died;
You lose his presence, but Heaven has gained a saint.

